# Protocol for a pilot randomised controlled clinical trial to compare the effectiveness of a graduated three layer straight tubular bandaging system when compared to a standard short stretch compression bandaging system in the management of people with venous ulceration: 3VSS2008

**DOI:** 10.1186/1745-6215-11-26

**Published:** 2010-03-09

**Authors:** Carolina D Weller, Sue Evans, Christopher M Reid, Rory Wolfe, John McNeil

**Affiliations:** 1 Department of Epidemiology and Preventive Medicine, School of Public Health and Preventive Medicine, Level 3 Burnet Building, The Alfred Hospital, Monash University, Melbourne, Australia

## Abstract

**Background:**

The incidence of venous ulceration is rising with the increasing age of the general population. Venous ulceration represents the most prevalent form of difficult to heal wounds and these problematic wounds require a significant amount of health care resources for treatment. Based on current knowledge multi-layer high compression system is described as the gold standard for treating venous ulcers. However, to date, despite our advances in venous ulcer therapy, no convincing low cost compression therapy studies have been conducted and there are no clear differences in the effectiveness of different types of high compression.

**Methods/Design:**

The trial is designed as a pilot multicentre open label parallel group randomised trial. Male and female participants aged greater than 18 years with a venous ulcer confirmed by clinical assessment will be randomised to either the intervention compression bandage which consists of graduated lengths of 3 layers of elastic tubular compression bandage or to the short stretch inelastic compression bandage (control). The primary objective is to assess the percentage wound reduction from baseline compared to week 12 following randomisation. Randomisation will be allocated via a web based central independent randomisation service (nQuery v7) and stratified by study centre and wound size ≤ 10 cm^2 ^or >10 cm^2^. Neither participants nor study staff will be blinded to treatment. Outcome assessments will be undertaken by an assessor who is blinded to the randomisation process.

**Discussion:**

The aim of this study is to evaluate the efficacy and safety of two compression bandages; graduated three layer straight tubular bandaging (3L) when compared to standard short stretch (SS) compression bandaging in healing venous ulcers in patients with chronic venous ulceration. The trial investigates the differences in clinical outcomes of two currently accepted ways of treating people with venous ulcers. This study will help answer the question whether the 3L compression system or the SS compression system is associated with better outcomes.

**Trial Registration:**

ACTRN12608000599370

## Background

Venous disease is the most common cause of leg ulcers [1,2]. The refractory nature of venous ulcers affects the quality of life and work productivity of those persons afflicted [3]. This, in combination with the high costs of long-term therapy, makes venous ulcers a major health problem in developed countries. Management of venous leg ulcers is based on understanding pathophysiologic abnormalities [4,5]. Compression increases ulcer healing rates compared with no compression. Multi-layered systems are more effective than single-layered systems[6] High compression is more effective than low compression but there are no clear differences in the effectiveness of different types of high compression[6].

The short stretch compression system is often used as a standard treatment in mainland Europe and Australia. It is an inelastic bandage that has minimal extensibility when applied[7]. The main disadvantage of short stretch bandages is that they tend to become loose after a few hours of wear time and tend to slip down the leg[8]. Short stretch bandage application also requires the skill of experienced health care professionals[7] whereas elastic tubular bandages can be applied by patients and carers[8].

Application of the 3L elastic tubular system is very simple; it can be applied in minutes and does not require a trained health professional. The short stretch bandage needs to be applied by a trained practitioner and takes longer to apply. In some instances if applied incorrectly can cause damage to underlying tissues on the lower limb. In view of recent literature [9] that has demonstrated that community nurses were unwilling to use compression bandages because they were uncertain of which type of compression bandage to use, and also how to apply bandages. The 3L elastic compression system may be more acceptable to nurses who apply compression. The 3L elastic system may also increase the number of patients in the community who are willing to wear compression. There is evidence that some patients with venous ulcers have increasing difficulty in complying to compression bandaging[10]. The reasons for and determinants of non-adherent behaviour to compression is multifactorial and include pain, discomfort and lack of valid lifestyle advice by healthcare professionals [11].

The aim of this study is to compare the efficacy and safety of two high compression bandages in healing venous ulcers in patients with chronic venous ulceration; graduated three layer straight tubular bandaging (3L) compared to standard short stretch (SS) compression bandaging. The 3L compression bandage is easy to apply, needs little, if any, training to apply to the limb, is well tolerated by patients and is cost effective.

### Venous leg ulcer Incidence

Chronic leg ulcers are leg ulcers which have not healed themselves in a four week period. Chronic leg ulcers affect 1% of the general population in Australia [4], and 3.6% of people older than 65 years [5].

Venous ulceration represents the most prevalent form of difficult to heal wounds and these problematic wounds require a significant amount of health care resources for their treatment. The incidence of venous ulceration is rising with the increasing age of the general population. The most common cause of lower extremity ulcers is venous insufficiency and accounts for nearly 80% of all ulcers [12,13].

Risk factors for development of venous ulcers include venous disease, obesity, immobility, phlebitis, family history of varicose veins, deep vein thrombosis, previous surgery for varicose veins and congestive cardiac failure. Up to 50% of patients with chronic venous insufficiency have a history of leg injury [12,13].

### Aetiology of venous ulcers

The pathophysiology of venous ulceration is controversial [13] however it is believed that ulcers result from venous occlusion or valvular incompetence and subsequent superficial venous hypertension [14]. Venous leg ulcers occur as a result of underlying venous disease where damage has occurred to the superficial deep or perforating veins. Although aetiology of venous ulceration is unclear, it has been suggested that ulceration results from increased intraluminal pressure in the capillaries. This results in fibrin deposition around the capillaries [15]. White blood cells are activated and release proteolytic enzymes that cause further tissue destruction. The alternative 'Trap' hypothesis proposes that fibrin and macromolecules eventually leak into the dermis where they bind with growth factors, making then unavailable for the tissue repair process.

The most recent theories about pathogenesis of venous ulcer have associated it with microcirculatory abnormalities and generation of an inflammatory response. The molecular and cellular profiles of chronic skin wound wounds are substantially different than found in acute healing wounds. Healing time is influenced by patient age, ulcer duration and ulcer area, but not by type of venous incompetence [15,16]

### Diagnosis of venous ulcers

In most instances, diagnosis of venous ulceration may be made using clinical criteria alone; however in 25% of cases leg ulcers will have mixed characteristics. The ankle-brachial index is a common vascular assessment to determine underlying aetiology although the gold standard in evaluating venous disease is considered to be colour duplex ultrasonography [17]. More commonly, the ankle-brachial index is measured using a Doppler to exclude arterial disease.

### Treatment of venous ulcers

The mainstay treatment of leg ulcers is sustained graduated high compression bandaging of the affected limb [18]. This acts by reducing the abnormally high pressure seen in the superficial veins and underlying lower limb swelling and oedema. Healing can be expedited through elevation of the affected limb, improved mobility, weight reduction, and improved nutrition. High compression bandaging systems may be delivered using either elastic or inelastic bandaging. A recent meta-analysis of bandaging systems found that multilayer compression bandages seemed to be superior to single-layer bandages in promoting ulcer healing [18]. Based on current knowledge the multi-layer high compression system is described as the current gold standard for treating venous ulcers. However, to date, despite our advances in venous ulcer therapy, no convincing low cost compression therapy studies have been conducted. And although we know that high compression is more effective than low compression there are no clear differences in the effectiveness of different types of high compression.

### Venous ulcer healing

Venous leg ulcer healing rates vary considerably. In wound centres providing graduated compression bandaging treatment for venous ulcers, healing rates vary from 25 to 50% after 12 weeks to 40% - 70% at 12 weeks [19] and after two years of compression therapy 20% of all chronic venous leg ulcers remain unhealed and even when healing does occur, approximately 56% will recur [3].

There are many reasons for variation in treatment times for venous ulcers. Chronic wounds that measure less than 10 cm^2 ^and are present for less than 12 months at first treatment are likely to be healed in 71% of patients within 24 weeks of treatment [20]. Conversely, chronic wounds that were greater than 10 cm^2 ^and have been present for more than 12 months, had only a 22% likelihood of being healed within the 24 week period. High compression is more effective than low compression but there are no clear differences in the effectiveness of different types of high compression [18].

### Economic and personal burden of venous ulcers

The impact of leg ulcers is felt both in physical suffering and reduced quality of life of those affected and in financial costs to the community [21]. Analysis performed more than ten years ago in Australia estimated that venous ulcers were responsible for about $400 million annually in health care costs. The high prevalence of venous ulcer has a significant socioeconomic impact in terms of medical care, days off work and reduced quality of life [4]. Treatment of venous ulcers is both time consuming and costly. Most of this cost is associated with the supply of dressings including multi layer bandages and community nurse visits [17].

The projected cost of management of venous ulcers is significant. Currently, up to 20 per 1000 individuals over the age of 80 have an active leg ulcer. One in eight Australians are aged over 65 years. By 2044 those aged over 65 years will account for one in four Australians. The expected number of people aged over 65 years living in western societies is anticipated to double within the next 40 years or so. Because the cost and resource implication of management of venous ulcers will cause considerable strain on the health system, strategies to improve management and cost effectiveness of this condition must be seen as a priority. Leg ulceration is a chronic condition, the prevalence of which is likely to increase as populations age [22]. Leg ulcers are reported as having an impact on virtually every aspect of daily life: pain is common, sleep is often impaired, mobility and work capacity tend to be restricted, personal finances are often adversely affected [23], and social activities are restricted due to fear of injury and negative body image [24].

Chronic venous ulcer healing remains a complex clinical situation and often requires the intervention of skilled, but costly multidisciplinary wound care teams. Venous leg ulcers that have been present for a prolonged period of time pose a substantial management challenge for clinicians. Traditional treatment options have often been ineffective or associated with several drawbacks. Although multi layer compression therapy is the standard care provided for management of venous leg ulcers, we are still unsure of the optimum level of compression needed to heal venous ulcers. There is a need to assess the healing efficacy of the two compression bandaging systems in the management of people with venous ulceration. The three layer compression system is cheaper alternative and may improve potential for patients to tolerate and comply with wearing compression bandages.

## Methods/Design

### Rationale for Conducting the Trial

This pilot study is being conducted to assess the healing efficacy and safety of a graduated three layer straight tubular bandaging system when compared to standard compression bandaging system in the management of people with venous ulceration. Indications of increased venous ulcer healing efficacy of the 3 layer compression system may provide a basis for greater compliance from patients and therefore improve healing rates. As an easy to apply, economical compression system this intervention may be a useful treatment for people with venous ulcers. We plan to recruit sufficient numbers at each site to test recruitment strategy and recruitment rate to inform a national study.

### Summarized Details of Investigational Products

The Investigational Product being studied is *Tubular - Form*; an elastic tubular bandage. Tubular form is listed with TGA (AUST L 59144) as a treatment for varicose conditions and control of oedema. In this study the investigational product will be used in graduated lengths of 3 layers of the tubular bandage system as follows:

1. from base of toes to just under knee (long layer)

2. from base of toes to above calf pump (medium layer)

3. from base of toes to mid gaiter (short layer)

The control group will be treated with standard short stretch compression therapy which consists of:

1. a padding layer

2. inelastic short stretch compression bandage

3. a tubular stocking

All layers of standard compression therapy will be applied from base of toes to just under the knee.

### Objectives

#### Hypotheses

##### Null Hypothesis

There is no difference in wound size reduction with graduated three layer straight tubular bandaging system when compared to standard short stretch compression therapy bandage.

##### Alternative Hypothesis

There is a difference in wound size reduction with graduated three layer straight tubular bandaging system when compared to standard short stretch compression therapy bandage.

#### Primary Outcome

The percentage reduction of wound area from baseline compared to week 12 following randomisation. The size of the wound area will be measured by Visitrak wound measurement system and wound digital photographs.

#### Secondary Outcomes

1. The proportion of ulcers healed within the trial period. (Complete healing is defined as full 100% epithelialisation or skin closure without drainage)

2. The incidence of treatment related adverse events

3. Reliability and acceptability (withdrawals)

4. Number of adverse effects and serious adverse events

5. Quality of Life measures at baseline, at end of treatment and at 3 month follow up (SF 36 and Cardiff Wound Impact Schedule (CWIS))

6. Self-reported compliance of compression bandage

7. Recurrence rates.

### Participant Selection

#### Type, Source and Number of Participants

Forty six participants will be recruited into the study from speciality wound clinics. Attendance at the clinics is by referral for clinical advice and management from General Practitioners or community nurses.

Study participants will be aged over 18 years of age, and will have a venous ulcer that is confirmed by CEAP clinical assessment to be the result of chronic venous insufficiency. Venous disease can be classified according to severity, cause and specific abnormality using the CEAP classification. Use of such a classification improves the accuracy of the diagnosis. The elements of CEAP classification are: **C**linical severity, **E**tiology or cause, **A**natomy and **P**athophysiology.

Participants may have multiple ulcers but only ONE will be considered to be the target ulcer. This will be the largest ulcer (between 1 cm^2 ^and 20 cm^2^) and separated from other ulcers by at least 2 cm.

Potential study participants will be provided with a study information sheet and asked to sign an Informed Consent Form. Following this, a formal assessment will be undertaken by a research nurse to determine eligibility according to the following inclusion and exclusion criteria.

##### Inclusion Criteria

Male and female study participants who meet all of the following criteria can be entered into the study:

1. Presence of a venous ulcer that is confirmed by CEAP clinical assessment to be the result of chronic venous insufficiency

2. Aged over 18 years

3. Present with clinical evidence of chronic venous insufficiency and chronic venous ulceration as evidenced by one or more of the following:

i. lower limb pigmentation

ii. varicose eczema

iii. lipodermatosclerosis

iv. varicose veins

4. Chronic venous leg ulcer (target ulcer) that

i. Has been present for at least 4 weeks

ii. is of an area equal to or greater than 1 cm^2 ^but less than or equal to 20 cm^2 ^as measured by digital planimetry techniques

5. Ankle Brachial Pressure index of equal or greater than 0.8 mmHg

6. Ankle circumference of greater than 20 cm and less than 30 cm

7. Mobile, and able to return for required treatments and study evaluations without undue hardship

8. Able to give Informed Consent

9. Able to understand and comply with the requirements of the trial

##### Exclusion Criteria

Study participants who meet any of the following criteria will not be eligible for participation in this study:

1. Unable or unwilling to wear compression bandage as directed

2. Evidence of severe liver disease, cardiac disease or chronic pulmonary disease

3. Medical condition likely to require systemic corticosteroids during the study period

4. Any significant condition that may preclude the participant from the study (e.g. severe depression or psychiatric illness)

5. Clinically suspected deep vein thrombosis

6. Unable or unwilling to attend clinic for routine treatment

7. Participation in this trial previously and/or who dropped out or were withdrawn

### Participant Consent

Informed consent will be taken according to ICH GCP (International Conference on Harmonization Good Clinical Practice). Before recruitment and enrolment into the study, each prospective participant will be given a full explanation of the nature and purposes of the study, and a copy of the Information Sheet to review. Once the essential study information has been provided, and the Investigator is assured that each study participant understands the implications of participating in the study, the participants will be asked to give consent to participate in the study by signing and dating the informed consent form A notation that written informed consent has been obtained, with date, will be made in the study participants source documentation and Case Report Form (CRF). The completed consent forms will be retained by the Investigator and a copy of the consent form will be provided to the study participant.

### Criteria for Participant Withdrawal

Participants will be advised that they may withdraw from the study at any time, for any reason, or if necessary, the Investigator may withdraw a study participant to protect their health. Study participants may also be withdrawn for not complying with study procedures such as not wearing compression bandages. The reasons for withdrawal will be fully documented in the study participants notes and CRF. Participants who are withdrawn or drop out of the randomised treatment will be allocated for continuing follow up care to the specialty wound clinic for wound management. All adverse events will be documented and the reasons for withdrawal ascertained wherever possible.

### Participant Compliance

During the treatment period participant's compliance will be assessed. Participants will be asked directly whether they have been wearing their bandage. Any episodes of non-compliance will be documented and the circumstances and duration recorded in the CRF. Should there be questions or consideration of deviation from the Protocol, clarification will be sought from the Study Coordinator. Any study participant treated in a manner that deviates from the Protocol, or who is admitted into the study but is not qualified according to the Protocol, may be ineligible for analysis.

### Randomisation

Randomisation will follow a computer generated allocation schedule (NQuery version 7), using allocation concealment to prevent prior knowledge of treatment assignment. Numbers will be assigned in strict chronological sequence and study participants will be entered in sequence. Randomisation in small block sizes (2 and 4) will be stratified by wound clinic site and wound size ≤ 10 cm^2 ^or >10 cm^2 ^(assessed from the wound tracings and digital photography). Each study participant will be allocated a unique randomisation number on successful completion of screening.

Patients will be randomised to receive either control or intervention group using a central computer generated random number generated by using a central computer generated randomisation sequence. The randomization code will be sent to the Investigator (or designee) who will prepare treatments according to the randomisation code.

To decrease bias and confounders the decision to accept or reject a participant will be made using inclusion and exclusion criteria. Informed consent will be obtained from participants prior to obtaining the randomisation code. Independent clinicians will assess the eligibility of the patient, input identifiers on the randomisation website interface to document eligibility and obtain the randomisation number and allocation of bandage type. The compression bandage systems will be applied according to the computer generated list allocation. The codes will only be revealed to the researchers once the recruitment, data collection and wound measurement analysis are completed. The allocation list will be stored within the Clinical Informatics and Data Management Centre of the DEPM.

### Plan and Trial Design

This pilot study is a randomised, multi-centre clinical trial to evaluate the efficacy and safety of graduated three layer straight tubular bandaging (intervention arm) compared with standard compression therapy (control arm) in participants with chronic venous ulceration. Neither participants nor study staff will be blinded to treatment. Screening evaluations will ensure that potential participants fulfil all requirements for entry into the study. Forty six study participants will be randomised (1:1) to either intervention arm or to the control arm of the study. Study participants will be reviewed weekly during the 12 week treatment period, and if healed in treatment period, participant will be also be reviewed monthly for three months during a subsequent follow up period.

In the following sections, a detailed description of all study procedures is given. All study visits and procedures are described and the evaluations performed at each visit are detailed. An overview and schedule of the visits and procedures is provided in Table 1.

**Table 1:** Study Schedule

		Screening Period		Treatment Period Weeks													Follow-Up Period Months		
	
		Screening visit		Day 1	1	2	3	4	5	6	7	8	9	10	11	12	Month1	Month2	Month3
Informed Consent		X																	

Inclusion Criteria		X																	

Exclusion Criteria		X																	

Randomisation		X																	

Medical history		X																	

Physical Examination		X														X	X	X	X

Vital Signs		X		X	X	X	X	X	X	X	X	X	X	X	X	X	X	X	X

Intercurrent Illness		X		X	X	X	X	X	X	X	X	X	X	X	X	X	X	X	X

Intercurrent Medication		X		X	X	X	X	X	X	X	X	X	X	X	X	X	X	X	X

Tolerability Assessment				X	X	X	X	X	X	X	X	X	X	X	X	X	X	X	X

Target ulcer assessment		X		X	X	X	X	X	X	X	X	X	X	X	X	X	X	X	X

SF 36 and CWIS				X												X			X

Adverse events				X	X	X	X	X	X	X	X	X	X	X	X	X	X	X	X

Compression hosiery fitting/ application		X														X^2^			

Check for Ulcer Recurrence																	X	X	X

Care of ulcer information sheet		X														X^2^	X^4^	X^4^	X^4^

Referred back to specialty wound clinic																X^3^	X^4^	X^4^	X^4^

X^2^: If healed.

X^3^: If not healed.

X^4^: If ulcer has recurred.

*: If target ulcer has completely healed, (defined as 100% epithelialisation with no presence of exudate or scab).

### Screening Evaluation

The screening period will last for one week during which time study participants will be required to wear compression bandages as randomised. Prospective participants will be informed about all aspects of the clinical study, including procedures, risks and benefits. Receipt of this information will be acknowledged for each study participant through the provision of written informed consent by the participant. A copy of the information sheet and consent form will be provided to each participant.

The following screening assessments will be conducted for each potential participant:

Informed consent; assessment of compliance with the inclusion and exclusion criteria; patient medical history; concomitant medications and treatments; physical examination and target ulcer assessment.

### Treatment Period - Day 1

Participants will be bandaged according to their random number allocation. The intact standard compression bandage should be removed and the target ulcer and limb thoroughly cleansed with tap water and soap-free wash. The wound may be debrided if deemed clinically necessary as sloughy and exudative wounds with unhealthy wound beds will take longer to heal. Wound debridement and exudate management is important in early stages to ensure adequate wound bed preparation.

The following assessments will be conducted for each participant: Target Ulcer Assessment, Temperature, pulse and blood pressure, intercurrent illness and medication, SF 36 and CWIS Quality of Life, Adverse Events and confirmation of next visit (7 days time).

### Treatment Period - Weeks 1 to 12

Participants will return to the study centre weekly following Day 1 for the following assessments: Check whether the wound has healed, Target Ulcer Assessment, Assessment of tolerability of product (very comfortable, comfortable, uncomfortable, very uncomfortable), Temperature, pulse and blood pressure, intercurrent illness and medication, Adverse Events, SF 36 and CWIS Quality of Life (Week 12 only)

### Treatment Period - Early Completion

Once a wound has been judged to be completely healed (defined as 100% epithelialisation with no presence of exudate or scab), participants will be referred for correct fitting of long-term compression hosiery to prevent recurrence of venous ulceration. Participants will also receive an education pamphlet as a resource on how best to prevent recurrence. Participants will proceed to the Follow-Up Period and will be followed up monthly for 3 months.

### Follow-Up Period - Month 1, 2 and 3

Participants whose target ulcer have been judged to be completely healed by/at 12 weeks of treatment will need to attend the outpatient department at the treating hospital to be assessed monthly (± 2 days) for 3 consecutive months during the Follow-Up Period to monitor safety and recurrence rates. If this is not possible the participants will be phoned at these intervals and asked about recurrence.

At each follow up visit the participant will have the following assessments performed:

◦ Check whether the wound remains healed.

◦ If the target ulcer has recurred ascertain:

- Date of recurrence

- Target Ulcer Assessment

- Determine if study participant has continued with compression hosiery treatment. If yes, assess tolerability of compression hosiery (very comfortable, comfortable, uncomfortable, and very uncomfortable). If no; reason why.

◦ Adverse Events. Any untoward medical occurrence in a trial participant even if it does not necessarily have a causal relationship with the treatment.

◦ Ulcer treatment - Participants will be referred to wound clinic care if the ulcer has not healed or has recurred in the follow up period.

◦ SF 36 and CWIS Quality of Life (Month 3 only).

Study participants whose ulcers have not healed by/at the 12 week visit will be released from the study into the care of a wound clinic. All care will be made to ensure that all adverse events are followed until resolution. Study schedule outlined in Table 1.

### Duration of Treatment Including Follow-Up

The ulcer will be dressed weekly with standard compression bandage for one week run in period. The treatment period is 12 weeks duration. All participants will be seen on a weekly basis by an experienced leg ulcer practitioner. Additional visits may be necessary if clinically indicated. Study participants randomised to the intervention group will be treated with graduated three layer straight tubular bandage system once per week for 12 weeks. Study participants randomised to the control group will be treated with standard compression bandage for 12 weeks.

Study participants whose ulcers heal before or at 12 weeks of treatment will be assessed monthly for 3 consecutive months during the Follow-Up Period to monitor for safety and target ulcer recurrence rates. Study participants whose ulcers have not healed at the 12 week visit will be released from the study and returned to the care of the speciality wound clinic. All care will be made to ensure that all adverse events are followed until resolution.

### Assessment Procedures

#### Physical Examination

A physical assessment of the participant will include demographics (gender, date of birth, height, weight, smoking and alcohol history), vascular assessment taking particular note of any signs and symptoms of chronic venous insufficiency, ankle mobility, vital signs (temperature, pulse and supine blood pressure) and Ankle-Brachial Index to assess the arterial supply of the target limb. This is a non-invasive procedure that involves measuring blood pressure on the foot and on the arm and is necessary prior to application of any compression bandage to exclude arterial disease. Participants will not be included in the trial if they have an Ankle Brachial index of less than 0.8.

#### Target Ulcer Assessment

The target ulcer should be identified from the entrance criteria (i.e. largest ulcer on limb and separated from other ulcers by at least 2 cm).

##### Visitrak wound tracing

The ulcer outline will be traced and the ulcer area calculated to determine ulcer size

##### Digital photo of ulcer

The ulcer will be photographed using a digital camera and image saved and sent to an independent assessor who will be blinded to treatment assignment. The target ulcer will also be photographed when complete epithelialisation is evident.

##### Percentage of Granulation Tissue

The ulcer will be assessed for quality of wound bed tissue (red <25%, red 25%-50%, red 50%-75%, >75%, hypergranulated, other).

##### Depth

The ulcer will be assessed for depth with Visitrak probe (Grade I, II, III, IV).

##### Surrounding Skin

The condition of the surrounding skin will be assessed (normal, dry, wet eczema, dry eczema, erythema, other).

##### Exudate level

The level of exudate will be assessed (copious, medium, minimal, nil).

#### SF36 and CWIS Quality of Life

Participants will be asked to complete the SF36 and CWIS Quality of Life surveys at screening visit and at completion of study treatment. A copy will be retained in the CRF.

### Statistical Methodology

Statistical summaries and analyses of data will be performed at Monash University.

### Sample Size and Analysis plan

Participants will be randomised to graduated three layer straight tubular bandaging system (3L) or to a standard short stretch compression bandaging system. From a previous study [25] we anticipate an average reduction of 20% in wound size from baseline to follow-up in the group that receives 3L. To detect with 80% power a difference in the mean percentage reduction of 50% in the group with standard compression versus 20% in the 3L group requires 18 participants per group. This assumes a two-sided z-test (alpha = 0.05) of mean log (Area2/Area1) where Area2 is the area of the wound at follow-up and Area1 is the area of the wound at baseline. Percentage reduction is defined as 100(Area2-Area1)/Area1 and percentage reductions of 20% and 50% correspond to a difference in means of 0.47 on the scale of analysis (log(Area2/Area1)). From a previous study (Vowden et al, 2007) we estimate the standard deviation of log(Area2/Area1) to be 0.5 in each group. If a wound has completely healed and Area2 = 0 then we will use log (min (Area2)/Area1) in analysis where Area2 = Area1/100 is the smallest observed wound area at follow-up among those participants whose wounds did not completely heal. A two-sided p-value of 0.05 will be considered to be statistically significant.

With 18 participants per group the study will have 55% power to address the secondary endpoint of complete healing if the effect of standard compression is to improve the rate of healing from 20% of participants fully healed (with 3L) to 60% with standard compression.

The analysis of the primary endpoint will be by z-test and linear regression if the distribution of log (A2/A1) is reasonably symmetric. It is unclear from previous studies whether this will be the case. If necessary the analysis of hypothesis 1 will be by non-parametric Wilcoxon rank-sum test. If major differences are evident in key variables (age, gender, ethnicity, BMI, smoking, ABPI, ankle and calf circumference, range of ankle mobility, past medical history- diabetes, anaemia, hypertension, past surgical history-hip surgery, venous surgery/ligation, DVT, medication history) between allocated groups at baseline, then the baseline values will be included as covariates in a second linear regression model for the primary outcome. Imbalance will be defined as a difference in means (on a log scale for variables following a skewed distribution) of ≥ 0.33 standard deviations or, for binary and categorical variables a relative risk of ≥ 2 (where prevalence is ≥ 10%).

Finally, 18 participants per group need to have wound area assessed at the end of the 12-week follow-up. To allow for loss-to-follow-up of 20% of participants between baseline and follow-up we will randomise 23 participants to each arm.

### Pilot study sample size justification

We will recruit 46 participants in total to enable the estimation of the between-person variability in percentage reduction in wound size from baseline to 12 weeks. There are insufficient previous studies to ascertain a likely value for this variability for use in power calculations for a fully powered trial.

The analysis principle for the primary outcome will be Intention-to-treat. The data will be analysed according to the treatment group to which participants are randomised even if they do not comply fully with their treatment. If participants drop out of the study they will be asked if they would still be prepared to return at the 12 week end of treatment schedule for target ulcer measurement.

### Data Management

Data management will be managed by Monash University. A Data Management Plan will be completed outlining the data management process prior to the collection and analysis of study data. Original on-screen Case Report Forms will be used when entering information into the computer database. Entered data will later be double-checked against original case report forms for accuracy. All case report forms and data checking records will be retained as permanent records of the study.

### Trial Approval and Conduct

#### Regulatory Approval

The Principal Investigator will ensure that this study is conducted in full compliance with the Protocol, the Declaration of Helsinki, the International Conference on Harmonization (ICH) guideline for GCP, Australian Therapeutic Goods Administration (TGA) regulations, and all other applicable local laws and regulations. Compliance provides assurance that the rights, safety, and well being of study participants are protected.

#### Ethical Considerations

The monitoring and safety guidelines are outlined in the Monitoring Guidelines for the study. This study will be carried out according to the Declaration of Helsinki, the Notes for Guidance on Good Clinical Practice (2000) (CPMP/ICH/135/95) and the ICH GCP Guidelines. The Protocol has approval from Alfred Health, Austin Health, Melbourne Health, Queensland Health and Monash University Human Research Ethics Committees (HREC)/Institutional Review Board (IRB). The Investigator will report study progress to the HREC/IRB as required or at intervals not greater than one year.

#### Trial Monitoring

The task of the Study Monitor is to guarantee the best conduct of the study through frequent contacts by phone and in person with the responsible Investigator and research nurse, in accordance with the Monitor's Operating Procedures, with the purpose of facilitating the work and fulfilling the objectives of the study. These site visits will enable the Monitor to maintain current, personal knowledge of the study through review of the records, comparison with source documents, and observation and discussion of the conduct of the study with the Investigator. Site monitoring will occur at 4-6 week intervals. Case Report Forms (CRF) will be reviewed and compared with source documents. Queries will be identified and a request for further information will be sought. The organisation, monitoring, supply of study materials and quality assurance of the present clinical study is the responsibility of Carolina Weller (Monash University PhD student). In order to ensure the accuracy of data, direct access to source documents by the representatives of both the Study Monitor and regulatory authorities is mandatory. Anonymity of the study participants will be maintained at all times.

## Discussion

The 3VSS2008 trial is a multicentre open label parallel group randomised trial to determine whether graduated three layer straight tubular bandaging system is more effective in mean percentage reduction of healing venous ulcers when compared to a standard short stretch compression bandaging system in the management of people with venous ulceration. This is a pilot trial to determine the recruitment rate and assess the variability in the percentage reduction of wound size in each bandaging group for use in sample size calculation for a fully powered trial. The results will inform the design of a larger trial that could provide more precise estimates of the efficacy of the intervention.

### Current study status

The study commenced recruitment in February 2009 and has recruited 25 participants. Patient follow up has commenced with 12 participants who have healed. Due to slow recruitment numbers two other sites have been approached to recruit for the study. Expected recruitment completion is July 2010.

## Abbreviations

3L: 3 layer tubular form elastic bandage; ABPI: Ankle Brachial Pressure Index; BMI: Body Mass Index; CWIS: Cardiff Wound Impact Schedule; GCP: Good Clinical Practice; HREC: Human Research Ethics Committee; ICH: International Conference on Harmonization; IRB: Institutional Review Board; SS: Short stretch inelastic compression bandage.

## Competing interests

The authors declare they have no competing interests. Sutherland Medical (SM) provided Tubular elastic bandages. The analysis and reporting of the study will be undertaken independently of SM.

## Authors' contributions

CW designed the study in collaboration with JM, CR, SE. CW drafted the protocol manuscript.

RW calculated sample size and provided statistical advice for study. All authors read and approved the final manuscript.
